# Context-dependent risk stratification using a tuberculin skin test induration threshold of ≥ 15 mm in school tuberculosis screening in Hangzhou, China: a historical cohort study

**DOI:** 10.1186/s41182-026-01034-1

**Published:** 2026-07-21

**Authors:** Xiangjie Meng, Yin Tang, Jun Shou, Nan Jiang, Yu Zhang

**Affiliations:** 1 Department of Infectious Disease Control, Yuhang District Center for Disease Control and Prevention (Hangzhou Yuhang District Health Inspectorate), 373 Yuniao Street, Liangzhu Subdistrict, Yuhang District, Hangzhou, 311113 Zhejiang China; 2 Department of Tuberculosis Control, Hangzhou Center for Disease Control and Prevention (Hangzhou Health Inspectorate), 568 Ming Shi Road, Jianqiao Subdistrict, Shangcheng District, Hangzhou, 310021 Zhejiang China; 3 Department of Tuberculosis Control, Zhejiang Provincial Center for Disease Control and Prevention (Zhejiang Provincial CDC), 630 Xincheng Road, Binjiang District, Hangzhou, 310051 Zhejiang China

**Keywords:** Adolescents, BCG vaccination, Contact investigation, Pulmonary tuberculosis, School screening, Tuberculin skin test

## Abstract

**Background:**

Larger tuberculin skin test (TST) reactions are associated with higher tuberculosis (TB) risk, but the operational value of the ≥ 15 mm threshold among adolescents who already have a TST induration ≥ 10 mm during routine school screening is less well characterised in high-BCG coverage settings. We assessed whether TST induration ≥ 15 mm provided incremental risk stratification information for secondary school students and interpreted this threshold across routine screening, outbreak contact and previously cured TB contexts.

**Methods:**

We conducted a historical cohort study using school-based TB screening and TB management data from Yuhang District, Hangzhou, China. The primary cohort included 6077 students aged 12–18 years who were screened between September 2023 and November 2025 with a baseline TST induration ≥ 10 mm and no active TB at screening. Students were classified as having a TST ≥ 15 mm or a TST 10–14 mm. The incidence of active pulmonary TB was ascertained by deterministic linkage with TB management systems. Students in the 2020–2022 TST ≥ 15 mm routine-screening descriptive cohort, school-outbreak contacts and previously cured TB patients were analysed descriptively and were not included in the primary inferential comparison.

**Results:**

In the primary cohort, four incident active pulmonary TB cases occurred among students with TSTs ≥ 15 mm (0.25%; 169.03 per 100 000 person-years), whereas none occurred among students with TSTs 10–14 mm. The adjusted incidence density ratio was 22.74 (95% CI 2.42–3015.62; P = 0.004), with wide uncertainty due to sparse events. The 2020–2022 TST ≥ 15 mm routine-screening descriptive cohort had a similarly low absolute incidence (128.64 per 100 000 person-years). In the outbreak contact cohort, the incidence among TST ≥ 15 mm contacts was 438.68 per 100 000 person-years; this estimate was interpreted descriptively because baseline definitions and preventive-treatment coverage differed.

**Conclusions:**

A TST induration of ≥ 15 mm was associated with a higher incidence of active pulmonary TB in students than a TST of 10–14 mm, but the absolute risk after routine screening remained low. The threshold should be interpreted as a context-dependent risk marker that can support, but should not replace, the assessment of exposure history, previous TB and time since screening.

## Background

Tuberculosis (TB) remains a major public health threat; an estimated 10.7 million people fell ill with TB worldwide in 2024 [1]. Adolescents require particular attention because prolonged school contact can facilitate transmission and delayed detection [2].

TB infection is a key precursor of disease, with approximately 5%-10% of untreated infected individuals developing active TB during their lifetime [3]. The tuberculin skin test (TST) and interferon-gamma release assays (IGRAs) detect immune sensitisation to *Mycobacterium tuberculosis* but cannot distinguish recent infection from remote infection or persistent immune memory. This distinction is particularly relevant in China because BCG vaccination is included in the National Immunization Program and is recommended as a single intradermal dose at birth [4]. In Zhejiang Province, a 2017 coverage survey among children aged 24–35 months reported high BCG uptake, with 92.0% documented coverage among children with immunization cards, 88.3% receiving BCG within 28 days after birth, and BCG scars observed in 97.1% of children assessed for BCG scarring [5]. Although IGRAs are more specific in BCG-vaccinated populations, cost and access constraints mean that TST remains widely used in many routine screening programmes [6–10].

The general association between stronger TST reactivity and subsequent TB risk is well recognised. The practical uncertainty for school screening programmes is narrower: whether the ≥ 15 mm threshold adds useful risk stratification information among students who already meet a lower positive threshold and how this signal should be interpreted when BCG vaccination, exposure intensity and previous TB can also influence TST reactivity.

TST thresholds and criteria for further assessment or preventive treatment vary by setting. The US and Canadian guidelines use risk-stratified cut-offs, with ≥ 10 mm considered positive for several increased-risk groups [11, 12]. Chinese school TB guidance uses ≥ 15 mm as a strong-reaction threshold, prompting further assessment and management [13], whereas some high-burden settings prioritise exposure history, contact status, age and clinical eligibility when tuberculosis preventive treatment is implemented [14, 15].

We used routinely collected school screening data to assess whether TST induration ≥ 15 mm adds risk stratification value among students with a baseline TST ≥ 10 mm. A 2020–2022 TST ≥ 15 mm routine-screening descriptive cohort, a school-outbreak contact cohort and a previously cured TB cohort were analysed descriptively to contextualise the interpretation of strong TST reactivity in a school setting with high BCG coverage.

## Methods

### Study design and data sources

We conducted a historical cohort study using the Tuberculosis Management Information System and the Yuhang District Public Health Emergency Intelligent Control System. Individual BCG vaccination records could not be batch extracted from the available systems and were therefore manually verified only in a sex- and school-stage-matched sensitivity subset. The requirement for informed consent was waived because this record-based analysis used an anonymised linked analytic dataset.

### Study population

#### Primary routine school screening cohort, 2023–2025

Students aged 12–18 years who were registered in the Yuhang District Public Health Emergency Intelligent Control System and who underwent TST screening between September 2023 and November 2025 were eligible. The analytic population was restricted to this age group because the district annual PPD/TST-based school screening programme targets secondary school students, not primary school students. In this age group, TST screening is operationally used to identify students who require further evaluation, whereas routine annual PPD/TST testing is not used for primary school-aged children in the local programme because PPD reactivity can be influenced by prior BCG vaccination and repeated annual testing is not considered appropriate for that age group. Elementary school-aged children were therefore outside the target population for the annual screening dataset and were not included in this analysis. We included students with complete baseline TST documentation and excluded those diagnosed with active TB at screening or within 42 days after screening [16] or those with a history of TB to reduce misclassification of prevalent disease. The final cohort included 6077 participants and 8658.28 person-years.

#### 2020–2022 TST ≥ 15 mm routine-screening descriptive cohort

We also analysed a 2020–2022 TST ≥ 15 mm routine-screening descriptive cohort. This cohort was included because, in the available 2020–2022 data extract, the recorded and extractable follow-up dataset contained students with strong TST reactions, whereas TST 10–14 mm data were not recorded in a form that could be extracted for analysis. The cohort was therefore used only as a descriptive temporal reference for absolute incidence after a ≥ 15 mm reaction and was not used to compensate for the small number of events in the primary cohort or to strengthen the primary adjusted comparison. Three screen-detected baseline active pulmonary TB cases, all in female junior high school students, were excluded, leaving 743 participants and 3109.51 person-years.

#### Contact cohort from a school TB outbreak

A separate cohort used TST screening data from contacts in a 2022 school TB outbreak in Yuhang District. Eligible participants were enrolled students with complete TST records and the same exclusions as the primary cohort. In total, 1455 participants contributed 5714.35 person-years.

#### Cohorts of previously cured TB patients

Individuals treated for TB during childhood or adolescence were selected from the Hangzhou Tuberculosis Management Information System (2004–2025) if they were diagnosed at age ≤ 18 years, had completed treatment with a clinical cure [17] and had at least one post-cure TST result. This small group was included only to contextualise the interpretation of strong TST reactivity after prior TB treatment. Specifically, it illustrates that a TST ≥ 15 mm response after clinical cure may reflect persistent immune memory rather than recent infection, new exposure or a higher short-term progression risk. The group was not used for statistical comparison with the primary school-screening cohort.

### TST administration and grouping

Trained healthcare workers performed all TST screening. Routine school and cured-patient screening was conducted annually from September to November, whereas contact screening was repeated during the outbreak. A 0.1 mL dose containing 5 international units (IU) of purified protein derivative (PPD) was administered intradermally, and the induration was measured 48–96 h after injection to the nearest millimetre; in accordance with Chinese school TB guidance, the average of the transverse and longitudinal diameters was recorded [13]. For the primary objective, students with TSTs < 10 mm were excluded; the routine cohort was grouped as having TSTs 10–14 mm or TSTs ≥ 15 mm, and the contact cohort was grouped as having TSTs < 15 mm or TSTs ≥ 15 mm.

At screening, the participants were assessed for pulmonary TB symptoms. In accordance with Chinese school TB guidance [13], those with suspicious symptoms or a TST ≥ 15 mm were referred for chest imaging and further evaluation. Students with TSTs of 10–14 mm were not routinely referred for chest imaging unless they had suspicious symptoms. This asymmetric evaluation pathway could increase case ascertainment in the TST ≥ 15 mm group relative to the TST 10–14 mm group and was considered a potential source of detection bias in interpreting incidence comparisons. In repeated contact screening, follow-up for the TST ≥ 15 mm group began when a TST ≥ 15 mm was first recorded, whereas follow-up for those never reaching this threshold began at the final screening. Because subgroup baselines differed, contact-cohort comparisons were descriptive and not used for causal inference.

Individuals with a TST ≥ 15 mm and no active TB were advised to receive preventive treatment at designated hospitals (3-month isoniazid-rifapentine regimen) [18]. No preventive treatment for tuberculosis was documented in the designated hospital records for the main TST ≥ 15 mm cohort, which may reflect non-acceptance, incomplete capture outside designated hospitals, or both. In the contact cohort, treatment completion, interruption and whether a TB case occurred in the same class were used descriptively.

### Outcome definition and follow-up

The primary outcome was incident active pulmonary TB, including bacteriologically confirmed and clinically diagnosed disease as defined by national criteria [17], which was diagnosed at designated TB hospitals. Cases were ascertained by deterministic linkage with national/municipal TB management systems using unique identifiers and demographic variables; similar surveillance-linkage approaches have been used in large TB cohort studies [19].

Follow-up began at baseline TST screening and ended at incident active pulmonary TB diagnosis or administrative censoring on 16 April 2026, whichever occurred first. Because migration and loss-to-follow-up records were incomplete, students without a recorded TB diagnosis were assumed to remain event-free until the administrative censoring date; differential censoring could therefore not be excluded. One baseline TB patient was excluded from the 2023–2025 cohort, and three were excluded from the 2020–2022 cohort. The cured patient cohort was used only for exploratory interpretation of post-treatment TST reactivity and was not included in the incidence comparison.

### Statistical analysis

Analyses were performed using R version 4.5.3 (R Core Team, R Foundation for Statistical Computing, Vienna, Austria). The incidence density was expressed per 100 000 person-years. Exact Poisson 95% CIs were used for groups with events, Hanley’s rule of three for zero-event upper bounds [20] and Clopper-Pearson CIs for proportions.

Two-sided exact Poisson tests were used in the primary routine cohort for the TST ≥ 15 mm versus 10–14 mm comparison. The 2020–2022 TST ≥ 15 mm routine-screening descriptive cohort was analysed descriptively; an exploratory calendar-period comparison with the 2023–2025 TST ≥ 15 mm cohort used an exact Poisson test. No formal TST-group comparison was performed in the contact cohort because subgroup baselines differed.

Firth-type bias-reduced Poisson regression was fitted via the brglm2 package, with log(person-time) as the offset and adjustment for sex and school stage [21]. Sex and school stage were included only as prespecified adjustment covariates for the primary TST-category comparison, and their coefficients were not interpreted as independent associations. Adjusted incidence density ratios (IDRs) and 95% confidence intervals (CIs) were obtained using profile likelihood.

Sensitivity analyses included variance specification via Huber-White robust standard errors, quantified the strength of unmeasured confounding using E-values for the adjusted IDR and its lower 95% CI, and assessed the BCG balance in a descriptive sex- and school-stage-matched subset via McNemar’s exact test. For E-value calculations, IDRs were used as approximations of risk ratios under the rare-outcome assumption.

For the cured patient cohort, Spearman correlation was used to assess TST reactivity against time from diagnosis to screening; the Mann–Whitney U test and Fisher’s exact test were used for exploratory group comparisons. The two-sided α was 0.05.

### Quality control

**Fig. 1:**
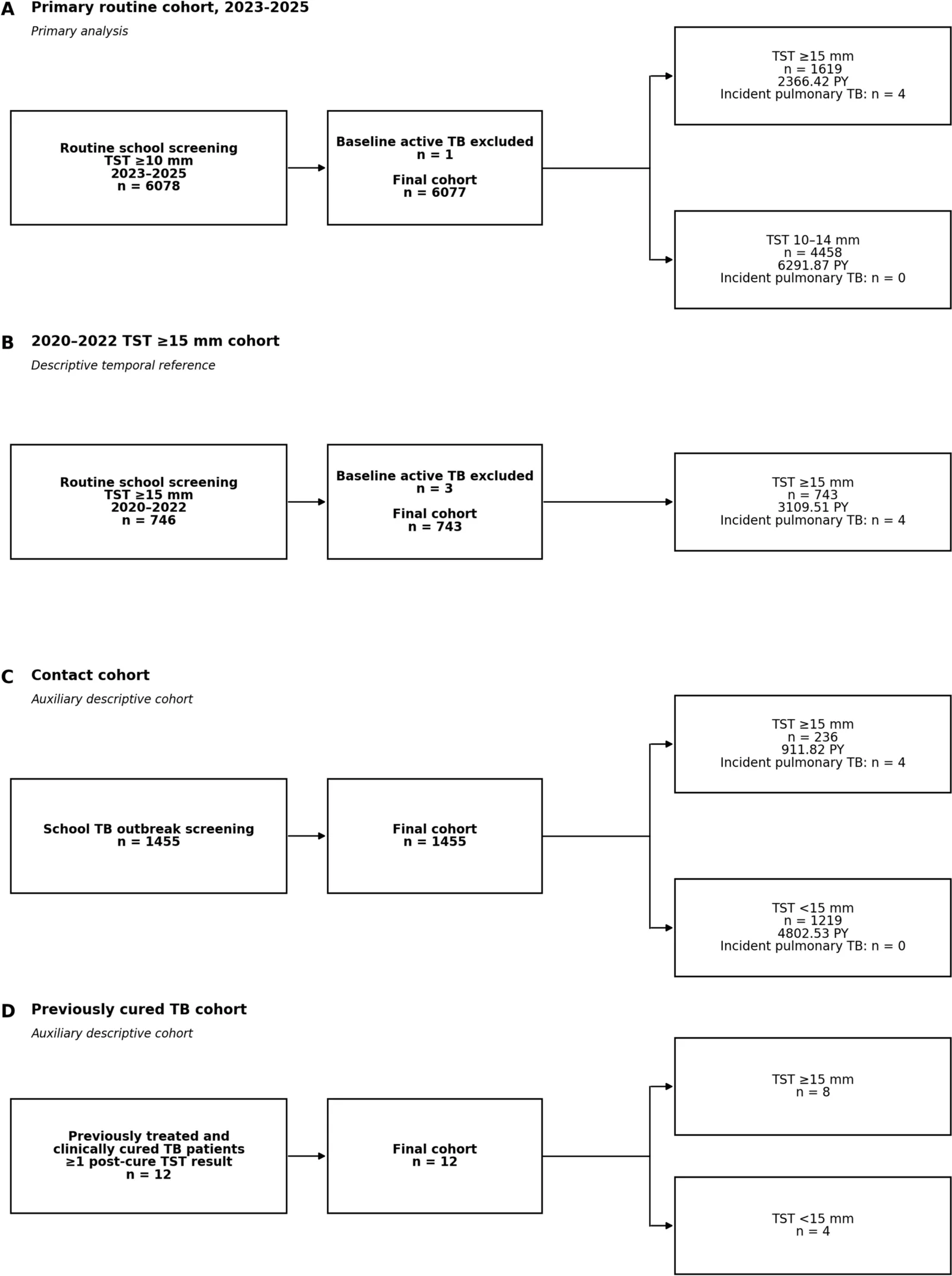
Study population selection and cohort structure. **A** Primary routine school screening cohort, 2023–2025. Among the 6078 students whose baseline TST induration was ≥ 10 mm identified through routine school screening, one female senior high school student who was diagnosed with active pulmonary tuberculosis at baseline screening was excluded, leaving 6077 participants (TST ≥ 15 mm, n = 1619; TST 10–14 mm, n = 4458). **B** 2020–2022 TST ≥ 15 mm routine-screening descriptive cohort. Among the 746 students with baseline TST induration ≥ 15 mm, three screen-detected baseline active pulmonary tuberculosis cases, all in female junior high school students, were excluded; the final 2020–2022 descriptive cohort included 743 students. **C** Contact cohort from a school tuberculosis outbreak. The final cohort included 1455 contacts, comprising 236 with TSTs ≥ 15 mm and 1219 with TSTs < 15 mm. **D** Previously cured tuberculosis cohort. Twelve previously cured tuberculosis patients with at least one post-cure TST result were included, including eight with TSTs ≥ 15 mm and four with TSTs < 15 mm. Person-years and incident active pulmonary tuberculosis counts are shown for the cohorts included in the incidence analyses. TB, tuberculosis; TST, tuberculin skin test; PY, person-years

Trained personnel entered the data via uniform definitions with range and logic checks, and a random 10% sample of records underwent independent double-entry verification. The study design and participant selection flowchart are shown in Fig. 1.

### Use of large language model (LLM)-assisted tools

During manuscript preparation, the authors used large language model (LLM)-based AI tools, namely Doubao and ChatGPT, for language editing, translation assistance, and readability improvement. These tools were not used to generate or alter study data, perform statistical analyses, interpret the results, select references, or determine the study conclusions. No LLM or other AI-assisted tool is listed as an author. All AI-assisted output was critically reviewed, verified, and edited by the authors, who take full responsibility for the accuracy, integrity, and final content of the manuscript.

## Results

### Routine school screening cohort

#### Baseline characteristics

After the exclusion of one baseline active pulmonary TB case among a female senior high school student, the final cohort included 6077 participants: 1619 with a TST ≥ 15 mm (2366.42 person-years) and 4458 with a TST 10–14 mm (6291.87 person-years). No preventive treatment for tuberculosis was documented in the designated hospital records for the TST ≥ 15 mm follow-up cohort. The groups differed mainly by sex; the school-stage distributions were similar (Table 1).

**Table 1:** Baseline characteristics and follow-up time of the primary routine cohort, 2023–2025

Characteristic	Total (N = 6077)	TST ≥ 15 mm (N = 1619)	TST 10–14 mm (N = 4458)	P value
Participants, n	6077	1619	4458	–
Person-years	8658.28	2366.42	6291.87	–
Follow-up time, median (IQR), years	1.48 (0.51–2.48)	1.48 (0.51–2.48)	1.48 (0.51–2.48)	0.582
Sex, n (%)				< 0.001
Female	3068 (50.5)	880 (54.4)	2188 (49.1)	
Male	3009 (49.5)	739 (45.6)	2270 (50.9)	
School stage, n (%)				0.640
Junior high school	4338 (71.4)	1163 (71.8)	3175 (71.2)	
Senior high school	1739 (28.6)	456 (28.2)	1283 (28.8)	

Values are n (%) unless otherwise indicated

Percentages are column percentages

P values were calculated using the Wilcoxon rank-sum test for follow-up time and Pearson’s χ^2^ test for sex and school stage. The 2020–2022 TST ≥ 15 mm routine-screening descriptive cohort was analysed separately and is not included in this table because the extracted dataset did not contain a contemporaneous TST 10–14 mm reference group. IQR, interquartile range; TB, tuberculosis; TST, tuberculin skin test

#### Association between TST ≥ 15 mm and TB risk

**Fig. 2:**
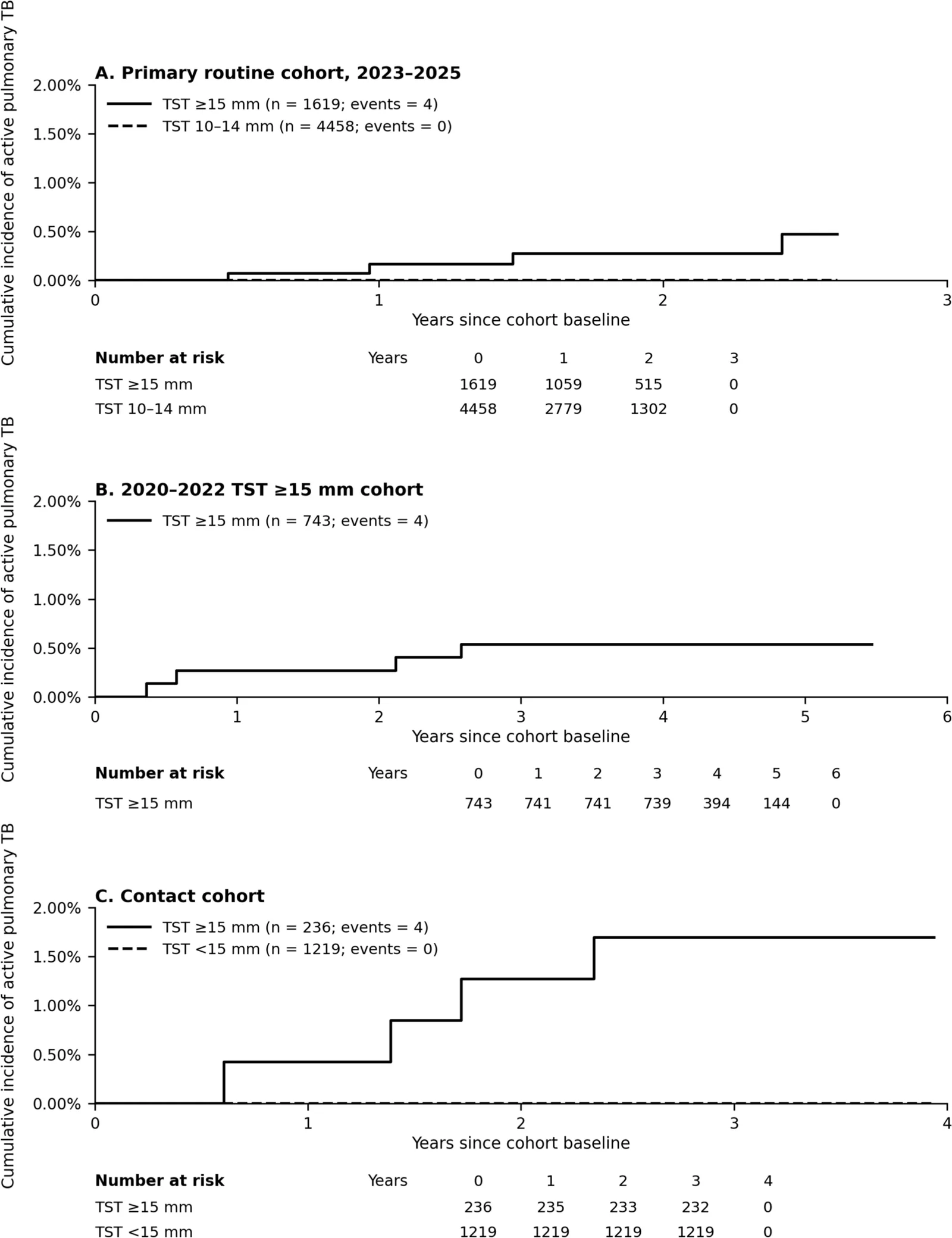
Cumulative incidence of active pulmonary tuberculosis after cohort baseline. Kaplan–Meier estimates of cumulative incidence are shown for **A** the primary routine cohort, 2023–2025, which compares students with TST indurations ≥ 15 mm and 10–14 mm; **B** the 2020–2022 TST ≥ 15 mm routine-screening descriptive cohort, after the exclusion of baseline active tuberculosis cases; and **C** the contact cohort from a school tuberculosis outbreak, which compares contacts with TST indurations ≥ 15 mm and < 15 mm. The numbers at risk are shown below each panel at yearly time points; a value of 0 at the final time point indicates that no participants remained under observation at or beyond that time point. The 2020–2022 cohort was analysed descriptively and was not included in the primary adjusted comparison. TB, tuberculosis; TST, tuberculin skin test

Four active TB cases occurred in the TST ≥ 15 mm group during 2366.42 person-years of follow-up; the median follow-up was 1.48 years. The crude cumulative incidence was 0.25% (4/1619), and the incidence density was 169.03 per 100 000 person-years (95% CI 46.06–432.79). No cases occurred in the TST 10–14 mm group, with an upper 95% confidence interval of 47.68 per 100 000 person-years estimated using Hanley’s rule of three. The exact Poisson test indicated a greater incidence rate in the TST ≥ 15 mm group than in the TST 10–14 mm group (P = 0.006; Table 2). Among the four incident cases, the median time from screening to diagnosis was 1.22 years (range, 0.47–2.42), and two cases occurred within one year (Fig. 2).

**Table 2:** Incident active pulmonary tuberculosis during follow-up by cohort and TST category

Cohort and TST category	Participants, n	Person-years	Incident active pulmonary TB, n	Crude cumulative incidence, %	Incidence rate per 100 000 PY (95% CI)	P value
Primary routine cohort, 2023–2025						
TST ≥ 15 mm	1619	2366.42	4	0.25	169.03 (46.06–432.79)	0.006
TST 10–14 mm	4458	6291.87	0	0.00	0.00 (0.00–47.68)	
2020–2022 routine-screening descriptive cohort						
TST ≥ 15 mm	743	3109.51	4	0.54	128.64 (35.05–329.36)	0.733^a^
Contact cohort						
TST ≥ 15 mm	236	911.82	4	1.69	438.68 (119.53–1123.20)	–
TST < 15 mm	1219	4802.53	0	0.00	0.00 (0.00–62.47)	–

Values are n unless otherwise indicated. Incidence rates are reported per 100 000 person-years. For zero-event groups, upper 95% confidence bounds were estimated using Hanley’s rule of three; all other 95% CIs used exact Poisson methods. P values were calculated using two-sided exact Poisson tests. The primary P value compares TST ≥ 15 mm with TST 10–14 mm in the 2023–2025 primary routine cohort. No P value was calculated for the contact cohort because the TST ≥ 15 mm and TST < 15 mm groups had different baseline definitions in repeated contact screening; therefore, this cohort was analysed descriptively. The 2020–2022 cohort was not included in the primary adjusted comparison because the extracted dataset did not contain a contemporaneous TST 10–14 mm reference group. CI, confidence interval; PY, person-years; TB, tuberculosis; TST, tuberculin skin test

a The P value compares the incidence rate in the 2020–2022 TST ≥ 15 mm routine-screening descriptive cohort with that in the 2023–2025 primary routine TST ≥ 15 mm group. This exploratory calendar-period comparison was descriptive and was not part of the primary TST-category comparison

Given the small number of events, no sex- or school-stage-specific inference was made.

#### Descriptive analysis of the 2020–2022 TST ≥ 15 mm routine-screening descriptive cohort

The 2020–2022 TST ≥ 15 mm routine-screening descriptive cohort included 743 students after the exclusion of three screen-detected baseline active pulmonary TB cases and contributed 3109.51 person-years. Four incident active pulmonary TB cases occurred, yielding a crude cumulative incidence of 0.54% and an incidence density of 128.64 per 100 000 person-years (95% CI 35.05–329.36; Table 2). The median follow-up was 4.45 years. This cohort was analysed as a descriptive temporal reference for students with TST ≥ 15 mm and was not used to supplement the event count for the primary 2023–2025 TST-category comparison. All the cases were female; three were senior high school students, and one was a junior high school student. The median time to diagnosis was 1.35 years (range, 0.36–2.58), with two cases occurring within one year.

The exploratory calendar-period comparison between the 2020–2022 and 2023–2025 TST ≥ 15 mm routine-screening cohorts did not reveal a statistically significant difference in incidence density (P = 0.733; Table 2). This comparison was descriptive and underpowered because only eight events occurred across both groups.

#### Multivariable analysis

Because the reference group had no events, maximum likelihood estimation was affected by separation. In the Firth-type bias-reduced Poisson regression adjusted for sex and school stage, a TST ≥ 15 mm was associated with a higher incidence rate than a TST 10–14 mm was but with substantial imprecision (adjusted IDR = 22.74, 95% CI 2.42–3015.62; P = 0.004; Table 3). The covariates were retained only for adjustment and were not interpreted as independent effects.

**Table 3:** Adjusted Firth-type Poisson regression estimate for the primary TST-category comparison

Characteristic	Category	Adjusted IDR (95% CI)	P value
TST category	TST 10–14 mm	Reference	–
	TST ≥ 15 mm	22.74 (2.42–3015.62)	0.004

CI, confidence interval; IDR, incidence density ratio; TB, tuberculosis; TST, tuberculin skin test

The model included TST category, sex and school stage, with log(person-time) as the offset

Sex and school stage were included only as adjustment covariates for the primary TST-category comparison and are not presented as independent associations

Estimates and 95% confidence intervals were obtained using Firth-type bias-reduced Poisson regression with profile penalised likelihood

### Descriptive observations of TB incidence among contacts with TSTs ≥ 15 mm

The contact cohort was analysed descriptively to contextualise TB incidence among TST ≥ 15 mm contacts in an outbreak-related setting where exposure intensity and preventive-treatment coverage differed from those of routine screening.

Among 236 contacts with TSTs ≥ 15 mm, 222 (94.1%) completed preventive treatment. Four incident active pulmonary TB cases occurred over 911.82 person-years, corresponding to a crude cumulative incidence of 1.69% and an incidence density of 438.68 per 100 000 person-years (95% CI 119.53–1123.20; Table 2). The median time to diagnosis was 1.55 years (range, 0.61–2.34), and three cases occurred within two years (Fig. 2).

All four incident cases among TST ≥ 15 mm contacts occurred in classes with at least one identified TB case. In this 128-student subgroup, three cases occurred among 124 students completing preventive treatment, and one occurred among two untreated students; none occurred among two students who interrupted treatment. Among 108 students from classes without identified TB cases, no active TB was observed.

For descriptive reference, no active TB cases occurred among the 1219 contacts with TSTs < 15 mm over 4802.53 person-years (Table 2). No formal P value was calculated for the contact cohort because the TST ≥ 15 mm and TST < 15 mm groups had different baseline definitions in repeated contact screening. Among the TST ≥ 15 mm contacts, the crude incidence density was 348.84 per 100,000 person-years in those completing preventive treatment, 2500.00 per 100,000 person-years in those not receiving preventive treatment and 0 per 100,000 person-years in those who interrupted treatment. Because events and person-time data are sparse, these data should not be used to infer treatment effectiveness.

### Exploratory analysis of TST reactivity among previously cured TB patients

Twelve individuals clinically cured of childhood or adolescent TB were included: five with culture-positive pulmonary TB, one with culture-negative pulmonary TB, four with extrapulmonary TB other than pleurisy and two with tuberculous pleurisy. The median age at diagnosis was 8.5 years, eight were male, and the median interval from diagnosis to TST screening was 5.23 years.

Overall, 66.7% (8/12; 95% CI 34.9–90.1%) had a TST ≥ 15 mm; among the four individuals tested > 10 years after diagnosis, three had a TST ≥ 15 mm. Among the two patients with repeated records, one remained ≥ 15 mm, and one increased from < 5 mm to 5–9 mm. The sample was too small for reliable association testing.

### BCG vaccination balance check and sensitivity analyses

In the 50 sex- and school-stage-matched pairs with manually verified BCG records, documented BCG vaccination status was concordant in 40 pairs: both students had documented BCG vaccination in 37 pairs, and neither student had documented BCG vaccination in 3 pairs. Ten pairs were discordant: documented BCG vaccination was present in the TST ≥ 15 mm student but not in the matched TST 10–14 mm student in 4 pairs, whereas it was present in the TST 10–14 mm student but not in the matched TST ≥ 15 mm student in 6 pairs. Overall, BCG vaccination was documented in 41 of 50 students in the TST ≥ 15 mm group and 43 of 50 students in the TST 10–14 mm group. This distribution did not suggest systematic imbalance in documented BCG vaccination between groups (McNemar’s exact test P = 0.754). Because individual BCG records were not batch-extractable for the full cohort, this analysis was a descriptive balance check rather than a definitive adjustment for BCG-related confounding.

In a sensitivity analysis using Huber-White robust standard errors, the TST-category point estimate was unchanged and remained significant (robust adjusted IDR = 22.74, robust Wald 95% CI 11.05–46.77; P < 0.001). Because sparse events and separation made the robust Wald interval much narrower than the primary profile-likelihood interval, this analysis was considered supportive only, and the Firth profile-likelihood estimates remained the basis for primary inference.

E-values were calculated by treating the IDR as a risk-ratio approximation under the rare-outcome assumption. The E-value was 44.97 for the adjusted point estimate and 4.27 for the lower profile-likelihood 95% CI limit. Thus, an unmeasured confounder would need a strong association with both TST category and TB incidence to fully account for the point estimate, but sparse events and the wide CI require cautious interpretation.

## Discussion

### Principal findings and contributions

This study does not attempt to re-establish the general principle that stronger TST reactions are associated with higher TB risk. Rather, it quantifies how the ≥ 15 mm strong-reaction threshold performed within a specific operational setting: routine school screening of students who already had TST induration ≥ 10 mm, supported by descriptive comparisons from outbreak contact and previously cured TB contexts.

Among students with a baseline TST ≥ 10 mm, TST ≥ 15 mm was associated with higher subsequent TB incidence than TST 10–14 mm in the adjusted model (adjusted IDR = 22.74; P = 0.004; Table 3), yet the absolute risk remained low in the crude analysis (0.25%; 169.03 per 100 000 person-years; Table 2). The 2020–2022 TST ≥ 15 mm routine-screening descriptive cohort showed a similarly low absolute incidence (128.64 per 100 000 person-years). Although the observed incidence rates in the TST ≥ 15 mm group were higher than the reported national pulmonary TB notification rates among Chinese children and adolescents [22], such comparisons are limited by differences in population, screening intensity and ascertainment.

In settings with routine BCG vaccination and limited individual BCG data, a TST ≥ 15 mm may be most useful as a trigger for closer assessment and short-term follow-up, not as the sole criterion for preventive-treatment decisions. This interpretation is consistent with the observed low absolute incidence and the wide profile-likelihood CI around the adjusted estimate.

### Context-dependent interpretation of TST ≥ 15 mm

TST reactivity may reflect recent infection, remote infection, intensified exposure, reinfection, persistent immune memory after previous TB, or nonspecific/BCG-related antigenic stimulation [23–25]. The previously cured cohort was included to illustrate this interpretive boundary. In this small group, 66.7% still had a TST ≥ 15 mm a median of 5.23 years after cure, which is consistent with paediatric guidance that infection tests may remain positive after treatment and should not be used as tests of cure [25]. These observations support considering prior TB history when interpreting a ≥ 15 mm threshold, but they require validation in larger cohorts.

In the outbreak-related contact cohort, exposure intensity and preventive treatment completion were high, and the incidence among those with a TST ≥ 15 mm was 438.68 per 100 000 person-years. This estimate should not be interpreted as an independent exposure-context effect because exposure, preventive treatment, baseline definition and unmeasured factors differed.

The contact cohort nevertheless underscores the importance of interpreting TST results in their epidemiological context. All four TST ≥ 15 mm contact cases occurred in classes with at least one identified TB case, which is compatible with an exposure gradient. The crude incidence rates were 348.84 per 100 000 person-years among students completing preventive treatment and 2500.00 per 100 000 person-years among untreated students; however, the untreated group contained only two students, so no treatment-effect inference was possible.

### Time window and low observed risk below 15 mm

No active TB was observed among students with TSTs of 10–14 mm in the main cohort or TSTs < 15 mm in the contact cohort. This suggests low short-term progression risk in these analysed groups, but it does not prove an absence of risk and should not be extrapolated to students with a TST < 10 mm, who were not included in the primary comparison. This interpretation should also be considered alongside the asymmetric evaluation pathway in routine screening.

Cases tended to occur soon after screening, although event numbers were too small to define the duration of excess risk. Half of the cases in both routine TST ≥ 15 mm cohorts occurred within one year, and three of the four contact cohort cases occurred within two years. This pattern is broadly consistent with evidence that progression risk is often highest soon after recent infection [16], supporting time-limited intensified monitoring without implying that later risk is absent.

### Implications for school TB screening programmes

The findings suggest a practical interpretive framework for TSTs ≥ 15 mm that considers the exposure context, prior TB history and time since screening. The same threshold may differ in meaning between routine screening and outbreak investigations; prior TB may produce persistent strong reactivity through immune memory, and early post-screening risk may be the most relevant monitoring window.

Because these signals are based on few events, they should be viewed as exploratory signals for future risk stratification studies rather than as validated clinical or public health decision frameworks.

### Limitations and future research

Limitations include the single-district historical cohort design, few events, limited generalisability, incomplete control of BCG history, behaviour, exposure intensity and preventive treatment outside designated hospitals, and possible detection bias. In routine screening, students with a TST ≥ 15 mm were referred for chest imaging and further evaluation, whereas students with TSTs of 10–14 mm were not routinely imaged unless they had suspicious symptoms. The zero-event finding in the 10–14 mm group may therefore reflect both lower underlying risk and less intensive surveillance.

Differences in preventive-treatment coverage and baseline definitions limit interpretation of the contact cohort, which may also be affected by asymmetric risk windows, including possible immortal-time-related bias introduced by different baseline definitions. For the 2020–2022 routine-screening descriptive cohort, TST 10–14 mm data were not recorded in a form that could be extracted for analysis, and the cured TB cohort was very small. Future multicentre prospective studies should include standardised TST readings, IGRAs or other biomarkers, detailed exposure assessments, complete preventive-treatment records and repeated post-treatment measurements.

## Conclusions

In this exploratory real-world historical cohort analysis, TST ≥ 15 mm was associated with higher active pulmonary TB incidence than TST 10–14 mm, but the estimates were based on very few events, and the absolute risk remained low. The additional 2020–2022 TST ≥ 15 mm routine-screening, contact and previously cured TB cohorts were descriptive and should be interpreted only as context for strong TST reactivity. The ≥ 15 mm threshold may inform risk stratification as one component of a broader assessment that includes exposure history, prior TB history and timing after screening, but it should not be used as the sole basis for preventive-treatment decisions. Prospective studies with more complete BCG, exposure, imaging and preventive-treatment data are needed to validate these findings.

## Data Availability

The datasets generated and/or analysed during the current study are not publicly available because they contain de-identified student screening and tuberculosis surveillance-linkage data subject to local data governance and privacy restrictions. Aggregated data supporting the findings are available from the corresponding author upon reasonable request and with permission from the Yuhang District Center for Disease Control and Prevention.

## Abbreviations

BCG: Bacille Calmette-Guérin
CI: Confidence interval
IDR: Incidence density ratio
IGRA: Interferon-gamma release assay
IQR: Interquartile range
PPD: Purified protein derivative
PY: Person-years
TB: Tuberculosis
TST: Tuberculin skin test
